# IgG4-related acute interstitial nephritis and the potential role of mCRP autoantibodies: a case report

**DOI:** 10.1080/0886022X.2019.1635493

**Published:** 2019-07-11

**Authors:** Lei Pu, Ping Zhang, Guisen Li

**Affiliations:** a Renal Department and Institute of Nephrology, Sichuan Academy of Medical Sciences and Sichuan Provincial People’s Hospital, Chengdu, China;; b School of Medicine, University of Electronic Science and Technology of China, Chengdu, China

**Keywords:** IgG4-related disease, acute interstitial nephritis, acute kidney failure, mCRP autoantibodies

## Abstract

**Background:** IgG4-related acute tubulointerstitial nephritis is a type of autoimmune-mediated interstitial nephritis. Recently, autoantibodies against modified C-reactive protein (mCRP) were found to play a pathogenic role in renal diseases through the formation of tubulointerstitial lesions. This is the first case report on the presence of mCRP autoantibodies in a patient with IgG4-associated acute tubulointerstitial nephritis.

**Case presentation:** A 70-year-old man was admitted with renal dysfunction and a medical history of bile duct stenosis, an inflammatory pancreatic mass, hypertension, and diabetes. On admission, laboratory tests showed higher than normal levels of serum creatinine and IgG4 and lower than normal levels of complements 3 and 4. In addition, the mCRP autoantibody levels were elevated, and the findings of kidney biopsy revealed interstitial nephritis with rich plasma cells in the renal interstitium. The patient was administered prednisone and cyclophosphamide therapy, which resulted in a rapid improvement in renal function.

**Conclusion:** IgG4-related autoimmune disease should be considered in the diagnosis of patients who have tubulointerstitial nephritis with multisystem involvement. Further, mCRP autoantibodies may be associated with IgG4-related tubulointerstitial nephritis and might be useful as a diagnostic marker of the disease.

## Background

Acute interstitial nephritis (AIN) is one of the frequent causes of acute renal failure, and AIN accounts for 15–27% of all renal lesions [1]. In fact, in two large-scale studies on a series of patients with AIN, all the patients presented with acute renal failure [2,3]. According to the main causative factors, AIN can be classified into three types: drug induced, infection related, and autoimmune mediated. Autoimmune-mediated AIN accounts for 15–25% of all AIN cases [4]. Some examples of autoimmune-mediated AIN are Sjogren’s syndrome, lupus nephritis, tubulointerstitial nephritis and uveitis (TINU), and IgG4-related tubulointerstitial nephritis (TIN).

C-reactive protein (CRP) is a member of the pentraxin family. Under conditions of altered pH, high urea concentration, or low calcium concentration, native CRP dissociates irreversibly into monomers, which are known as mCRP. The mCRP monomers undergo conformational rearrangements to form isomers with distinct antigenic and physiochemical characteristics. mCRP is considered as the tissue and/or cellular form of acute reactive protein [5]. mCRP not only plays a role in the safe clearance of apoptotic material, but also provides protection against unwanted complement activation in the fluid phase [6]. Thus, autoantibodies against mCRP might affect the clearance of apoptotic materials and increase the risk of abnormal immunization against autoantigens. Recently, it was demonstrated that mCRP autoantibodies are highly prevalent in patients with TINU; further, mCRP autoantibodies are also associated with disease activity and treatment effectiveness in patients with TINU [7]. In addition, high levels of mCRP autoantibodies have been found in patients with lupus nephritis who have tubulointerstitial lesions [8]. However, the involvement of mCRP autoantibodies in IgG4-associated TIN has not been reported until now. In the present study, we report the first case of IgG4-related TIN in which the patient was positive for antibodies against modified C-reactive protein (mCRP).

## Case presentation

A 70-year-old man was admitted to the hospital because he had been suffering from edema of the lower extremities for 1 month. He had a 5-year history of renal dysfunction, bile duct stenosis, and an inflammatory pancreatic mass. Additionally, 5 years prior to the presentation, he had a baseline serum creatinine level of 200 μmol/L. At that time, the patient had undergone choledochal stent implantation. Three years ago, the stent had been removed, and he had begun to take oral prednisone 30 mg/day. The bile duct stenosis and pancreatic mass had been successfully eliminated, so the prednisone treatment had been discontinued 3 months prior to presentation. In the past month, he had experienced rapid progression of renal function. His medical history also included hypertension and diabetes. With the exception of bilateral lower limb edema, the findings from the physical examination conducted at the time of presentation were unremarkable.

Laboratory tests showed that his white blood cell count was 5.06 × 10^9^/L; hemoglobin, 87 g/L; platelet count, 126 × 10^9^/L; **CRP**, 30.23 mg/L; **erythrocyte sedimentation rate**, 80 mm/H; serum nitrogen, 32.79 mmol/L; **serum creatinine**, 555.8 μmol/L; and serum albumin, 32.7 g/L. He had normal liver function with normal amylase and lipase levels. **Urine analysis** showed that the protein level was 2+; red blood cell count, 80/μL; urine gravity, 1.010; urinary albumin/creatinine ratio, 330; **24-h urine protein**, 0.98–1.22 g; and **urine neutrophil gelatinase-associated lipocalin (NGAL)**, 236 ng/mL.

Immunological tests showed that the concentrations of **serum C3** (complement 3) and **serum C4** (complement 4) were lower than normal at 0.355 and <0.066 g/L respectively. Further, the patient had polyclonal hypergammaglobulinemia, as the total serum immunoglobulin G (IgG) level had increased to 31.7 g/L. However, the serum IgA and IgM concentrations were normal. The patient was negative for anti-self-antibodies, including antinuclear antibody, anti-double-stranded antibody, anti-Sjogren’s syndrome A antibody, anti-Sjogren’s syndrome B antibody, anti-proteinase 3, and anti-myeloperoxidase anti-neutrophil cytoplasmic antibody. Analysis of the **IgG subtypes showed** that the serum IgG1 level and the serum IgG4 level were obviously elevated at 23.7 g/L (4.90–11.40) and 10.90 g/L (0.08–1.40), respectively. **The mCRP autoantibodies level was also elevated at** 64.25%.

Kidney ultrasound examination showed that both kidneys were of normal size (10.6 × 5.2 cm and 10.6 × 5.6 cm), with uniform echo frequency and clear corticomedullary boundaries; moreover, there were no mass lesions. Chest computed tomography showed that there were multiple nodules in the both lungs. The abdominal radiography findings were unremarkable.

**Figure 1.**
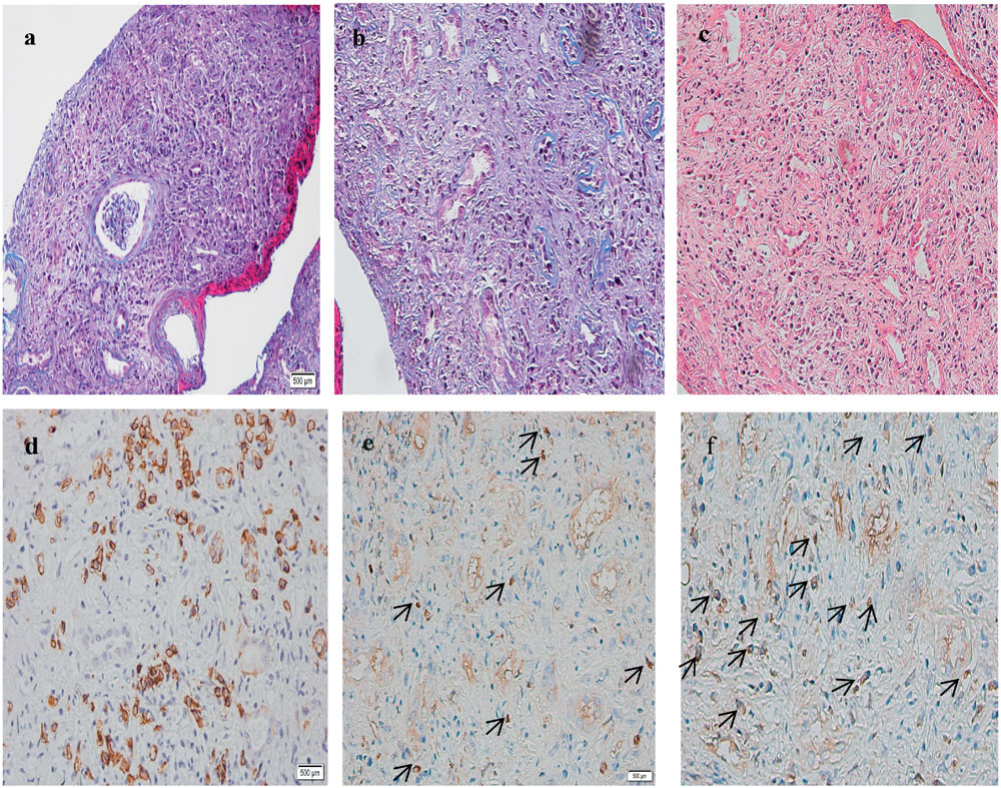
Pathological features of the renal biopsy specimen. (a) The tubulointerstitium shows marked injury, but the glomerulus shows no specific features (Masson trichrome staining, ×100). (b) Storiform fibrosis with tubular atrophy can be observed in the tubulointerstitium (Masson trichrome staining, × 200). (c) A mass of inflammatory cells, including lymphocytes and plasma cells, are seen infiltrating the interstitium (hematoxylin-eosin staining, ×200). (d) Immunohistochemical staining shows mostly CD138-positive plasma cells in the interstitium. (e, f) Immunohistochemical staining shows seven and thirteen IgG4+ and IgG + plasma cells in the same area of serial sections, respectively (Figures 1(e,f)) (indicated by the arrows). So the IgG4+/IgG + plasma cells ratio is calculated to be 53%.

The renal biopsy specimen exhibited severe tubulointerstitial changes, but the glomerulus shows no specific finding (Figure 1(a)). In particular, storiform fibrosis (Figure 1(b)) and inflammatory cells infiltration including plasma cells (Figure 1(c)) were marked in the interstitium. Immunofluorescence staining revealed that IgM, IgA, and C3 were present at low levels in the granular mesangial area. Electron microscopy demonstrated that there were no electron-dense deposits in the glomeruli. Immunohistochemical staining showed that most plasma cells in the interstitium were positive for CD138, and there were seven IgG4-positive plasma cells and thirteen IgG-positive plasma cells in the same area of serial sections (Figures 1(d–f)). So, the percentage of IgG4-positive plasma cells among the total IgG-positive plasma cells was 53%.

**Figure 2.**
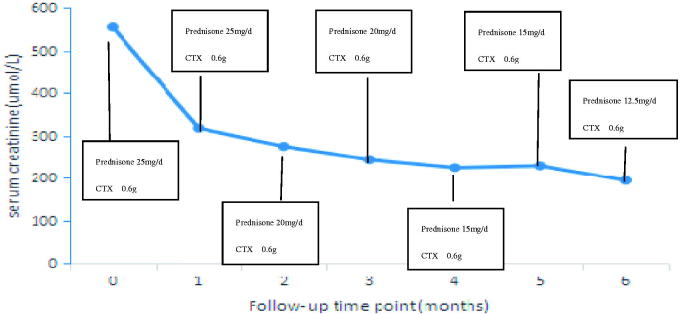
Changes in renal function during the follow-up, as indicated by the serum creatinine level.

We prescribed 25 mg/day oral prednisone and 0.6 g of cyclophosphamide (CTX) every month by intravenous infusion. The patient had been followed up for 6 months at the time of writing this paper. In the most recent follow-up examination, the serum creatinine level had decreased to 194 μmol/L, the serum C3 and serum C4 levels had increased to 0.668 and 0.209 g/L, respectively, and were close to normal, the serum IgG1 level was normal at 10.2 g/L, and the IgG4 level had considerably decreased to 3.72 g/L. Therefore, the dose of prednisone was decreased to 12.5 mg/day, and the cumulative dose of CTX was 3.6 g until to the time of writing this paper. Changes in renal function, as indicated by the serum creatinine level, during the follow-up are depicted in Figure 2.

## Discussion

We present here the diagnosis and treatment of a case of IgG4-related TIN, which is the first such case to be reported in which the mCRP autoantibody levels were elevated.

The findings from kidney biopsy were indicative of TIN. Further, based on the immune-related findings, including hypocomplementemia, and the increased level of mCRP autoantibodies, the TIN can be classified as autoimmune-mediated TIN. Since the patient was negative for anti-self-antibodies spectrum indicators were negative, there was no evidence for Sjogren’s syndrome or lupus nephritis. Further, ophthalmologic examination did not reveal anterior uveitis, so TINU was also ruled out. However, the patient in the current case presented with multiorgan involvement, including bile duct stenosis, a pancreatic inflammatory pseudo-tumor, pulmonary nodules, and decrease in renal function. Further, kidney biopsy was indicative of storiform fibrosis in the tubulointerstitium and dense infiltration of CD138-positive plasma cells, with the percentage of IgG4-positive plasma cells among the total IgG-positive plasma cells being >40%. In accordance with these findings, the serum concentration of IgG4 was significantly increased and was 10.9 g/L. Based on these findings and the diagnostic criteria of IgG4-related renal diseases proposed by Kawano et al. in 2011 [9], the patient was diagnosed with IgG4-related TIN.

In the present patient, the serum mCRP autoantibodies concentration had significantly increased. Similarly, elevated serum mCRP autoantibodies levels have been reported in TINU [7] and systemic lupus erythematosus with renal involvement [8,10]. Further, mCRP autoantibodies have been reported to play a pathogenic role in lupus nephritis by influencing the interaction between mCRP and C1q or complement factor H and regulating complement activation [11,12]. In the current case of IgG4-related TIN, we also detected diminished levels of complements three and four. This might indicate the pathologic involvement of mCRP autoantibodies in IgG4-associated TIN. However, although this case makes an important contribution to what is known about mCRP autoantibodies and IgG4-related renal disease, more investigations are required to determine whether the mCRP autoantibodies also play a pathogenic role in IgG4-related TIN by regulating the complement pathways, as it does in other autoimmune diseases.

Corticosteroids are considered as the first line of therapy for renal injury in IgG4-related TIN [13]. In this case, we found that his renal dysfunction was improved rapidly after 1 month of prednisone (25 mg/day) therapy, and it continued to improve with low-dose prednisolone over the next 6 months. In agreement with our observation, a retrospective study on 44 patients with IgG4-related TIN from Japan reported that a moderate initial dose of prednisolone (≤0.6 mg kg^−1^ day^−1^) was enough for rapid improvement of renal function, and renal function was maintained in the long term (>36 months) with low-dose prednisolone (the mean maintenance dose was 4.9 mg daily) [14]. In the present case, CTX was also administered. However, there is no indication about whether the addition of the immunosuppressant had a therapeutic effect, and there are no such reports in the literature too. Therefore, the potential therapeutic effect of concomitant treatment with immunosuppressants in IgG4-associated TIN should be investigated in the future.

## Conclusion

We report here a case of AIN and multiple organ involvement that was diagnosed as IgG4-related TIN at 5 years from its onset. Thus, the possibility of IgG4-associated disease should be considered in cases that present with TIN and multisystem involvement. Further, the anti-mCRP autoantibody level might be useful as a diagnostic indicator of the disease.
